# Supplementary material for: Optimization of Porphyran Extraction from Pyropia yezoensis by Response Surface Methodology and Its Lipid-Lowering Effects
Source: Mar Drugs. 2021 Jan 23;19(2):53. doi: 10.3390/md19020053 (PMC7911723; doi:10.3390/md19020053)
Supplement: Supplementary file 1 [file marinedrugs-19-00053-s001.zip › marinedrugs-1045296-supplementary/Supplementary file/Supplementary Figure 1-3.docx]

**Supplementary Figure**


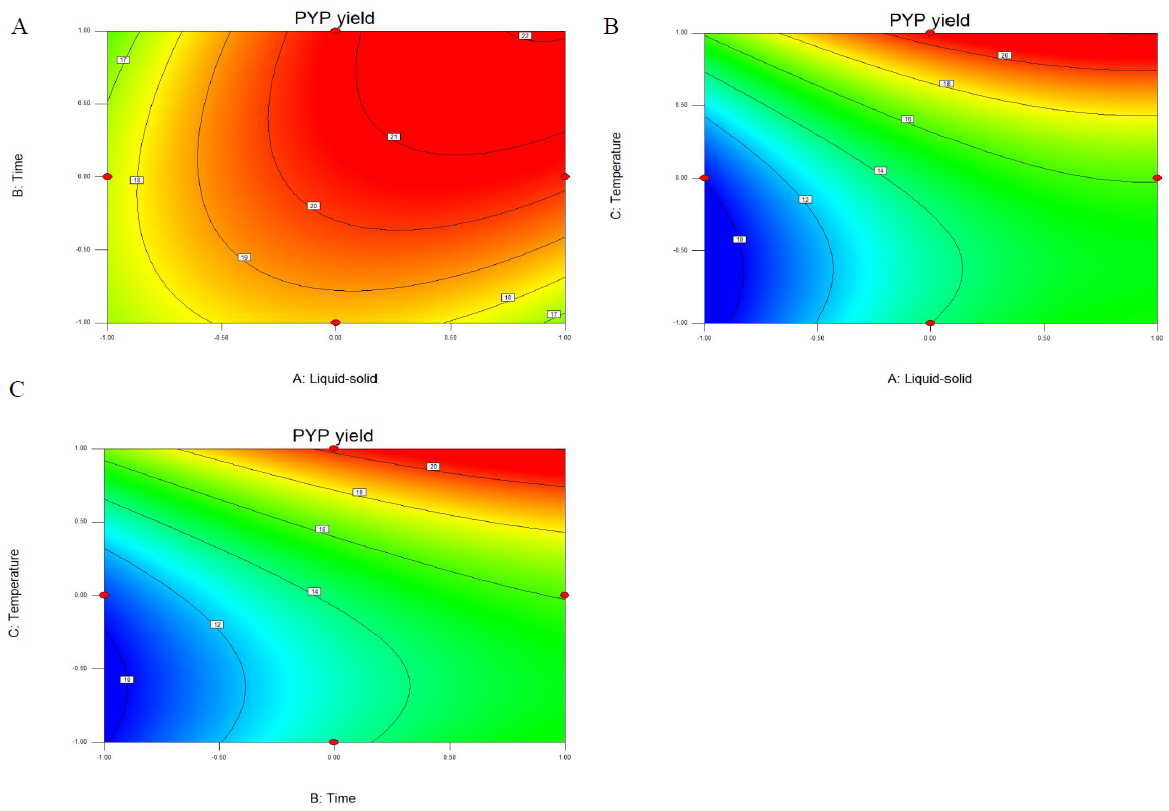


**Supplementary Figure 1.** 2-D contour plots of the effects of the various parameters on the PYP yield. (A) Liquid-solid ratio and time; (B) Liquid-solid ratio and temperature; (C) Time and temperature.

**
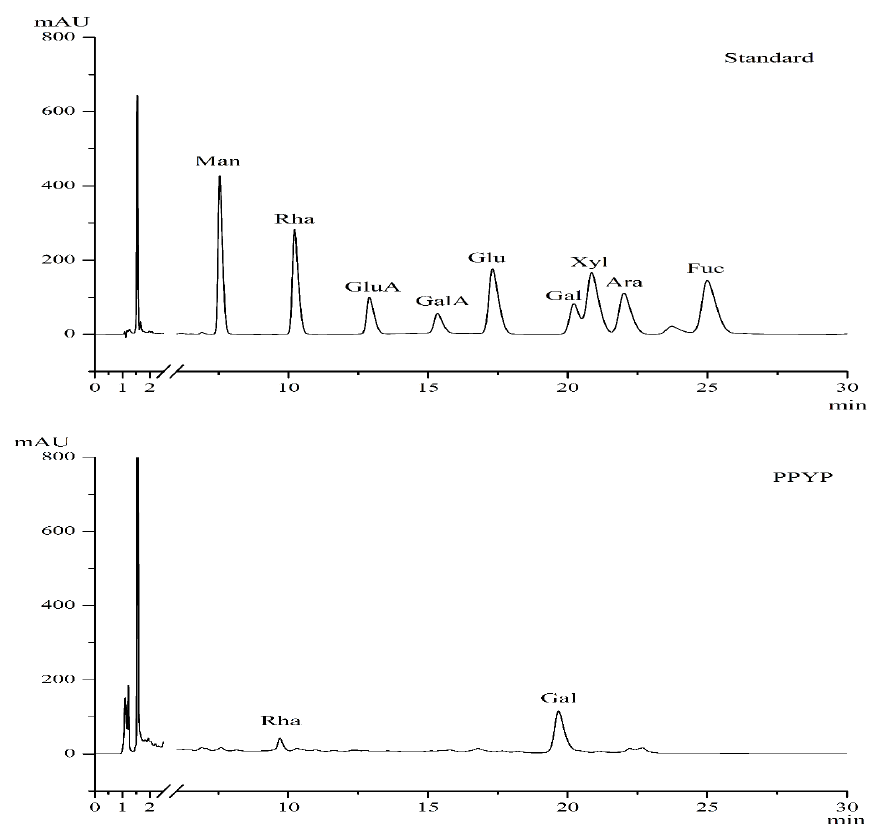
**

**Supplementary Figure 2.** Monosaccharide composition of PPYP.

**
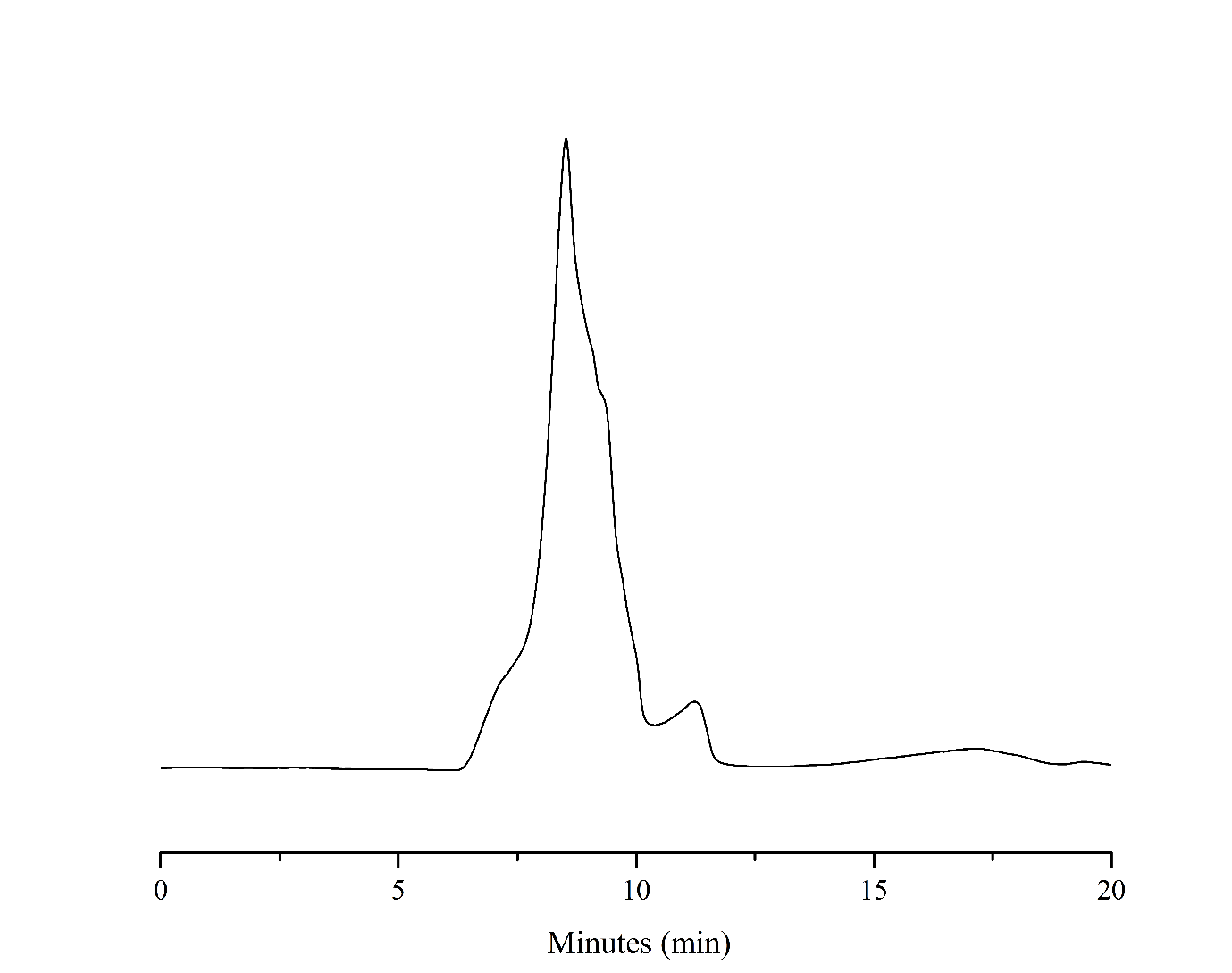
**

**Supplementary Figure 3.** The molecular weight determination of PPYP.
